# Glucagon-like peptide-1 receptor agonists for the management of diabetic peripheral neuropathy

**DOI:** 10.3389/fendo.2023.1268619

**Published:** 2024-01-19

**Authors:** Chunyan Liu, Tianqiang Wu, Na Ren

**Affiliations:** ^1^ Department of Endocrinology, First Affiliated Hospital, Heilongjiang University of Chinese Medicine, Harbin, China; ^2^ Department of Integrated Traditional Chinese and Western Medicine, Heilongjiang University of Chinese Medicine, Harbin, China

**Keywords:** diabetic peripheral neuropathy, glucagon-like peptide-1 receptor agonists, neuroprotection, inflammation, oxidative stress

## Abstract

Diabetes mellitus is a prevalent chronic disease characterized by hyperglycemia. Diabetic peripheral neuropathy (DPN) is one of the complications of diabetes mellitus and is caused by neuron injury induced by hyperglycemic circumstances. The incidence of DPN varies among different countries and regions, ranging from nearly 20% to over 70%. Patients with DPN may encounter symmetric pain or discomfort of the extremes, leading to reduced quality of life and even amputation. The pharmacological management for painful DPN mainly includes antidepressants due to their analgesic effects. Nevertheless, effective therapies to impact the pathogenesis and progression of DPN are lacking. Glucagon-like peptide-1 receptor (GLP-1R) agonists show efficacy in controlling blood glucose and serve as a treatment modality for diabetes mellitus. In recent years, evidence has been proposed that GLP-1R agonists exert neuroprotective effects through modulating inflammation, oxidative stress, and mitochondrial dysfunction. On the other hand, clinical evidence on the potential of GLP-1R agonists for treating DPN is still controversial and limited. This narrative review summarizes the preclinical and clinical studies investigating the capacity of GLP-1R agonists as therapeutic agents for DPN.

## 1 Introduction

Diabetes mellitus (DM) is a chronic disease of the endocrine system characterized by hyperglycemia mainly induced by insufficient insulin secretion or insulin resistance (1). It is estimated that over 500 million individuals are affected by this disease in 2021, and the prevalence of DM will be 1.3 billion by the year 2050 (2). The incidence of DM is dramatically increasing in low- and middle-income countries. In China, it has been reported that 12.4% of adults are affected by DM, which accounts for approximately a quarter of DM patients worldwide; by 2045, it is estimated that there will be 174 million DM patients in China (3). Patients with DM may encounter thirst, frequent urination, weight loss, and other symptoms; moreover, complications of DM may occur, such as diabetic retinopathy, diabetic nephropathy, cardiovascular diseases, and diabetic neuropathy (4–6). These complications put patients with DM at a high risk of low quality of life, increasing treatment cost, disability, and even mortality (7, 8). Diabetic peripheral neuropathy (DPN) is the most common type of diabetic neuropathy (9). It is estimated that in China, approximately half of patients with DM are complicated with DPN (10). Patients with DPN would likely experience symmetric pain or discomfort, typically presenting as numbness, burn-like or sting-like pain, and the feeling of wearing stockings or gloves (4). These symptoms may severely decrease the quality of life and induce anxiety and depression in patients (11). Unfortunately, treatments for DPN are still largely insufficient.

Glucagon-like peptide-1 (GLP-1) is a peptide secreted by the intestinal tract after the stimulation of food intake, which induces the secretion of insulin by β cells, thus modulating blood glucose (12). Based on this mechanism, GLP-1 receptor (GLP-1R) agonists have been developed for the treatment of DM (13). Until now, various clinical trials have demonstrated that GLP-1R agonists show favorable efficacy and tolerable safety in lowering blood glucose in patients with DM (14–16). Apart from the glycemic-lowering effect, GLP-1R agonists are considered pharmacological options for treating obesity and other complications of DM, such as cardiovascular diseases and diabetic nephropathy (17–19). Notably, evidence has been proposed that GLP-1R agonists present neuroprotective effects (20–22). Several clinical studies have also indicated that GLP-1R agonists may serve as a potential therapeutic strategy for DPN (23–25).

This narrative review aimed to summarize the currently available perspective on the potential involvement of GLP-1R agonists in DPN and shed light on the future direction of clinical studies.

## 2 Current perspective on DPN

### 2.1 Prevalence of DPN

The prevalence of DPN varies among different countries and regions. A cross-sectional study conducted in two cities of middle China reported that the overall prevalence of DPN was 71.2% in patients with DM (26). Another study conducted in the southern region of China indicated that the overall prevalence of DPN is 33.1% in patients with type 2 DM who are overweight or obese (27). In Taiwan, the prevalence of DPN is 21.3% (28). A systematic review and meta-analysis involving 29 studies from 8 countries found that in Latin America and the Caribbean region, the incidence of DPN is 46.5% (95% confidence interval: 38.0-55.0%) (29). In the Middle East, a study performed in Saudi Arabia reported that in patients with type 2 DM, the prevalence of DPN assessed by the Michigan Neuropathy Screening Instrument (MNSI) is 27.7% (30). Another study conducted in Jordan showed that the incidence of DPN is 36% in patients with DM (31). In the United States, a study assessed 11.9 million adults with DM and found that a total of 3.9 million (32.7%) patients have symptomatic DPN (32). Another study performed on youth (age < 20 years) reported that the prevalence of DPN assessed by the MNSI is 7% in youth with type 1 DM and 22% in youth with type 2 DM (33). The Diabetes Control and Complications Trial/Epidemiology of Diabetes Interventions and Complications (DCCT/EDIC) study evaluated the incidence of DPN in participants with type 1 DM, followed for over 23 years and illustrated that the incidence of DPN is 33% (34). In Europe, a study randomly assessed 3,250 patients with DM from 31 centers in 16 European countries and suggested that the overall prevalence of DPN is 28% without significant geographical differences (the EURODIAB IDDM Complications Study) (35). The KORA F4/FF4 Study conducted in Germany reported a prevalence of DPN of 25% (36). The ADDITION Demark study reported that the prevalence of DPN was up to 34.8 (37). Generally, the prevalence of DPN in patients with DM ranges from nearly 20% to over 70% in different countries and regions, where studies in China reported the highest prevalence of DPN in patients with DM, suggesting the urgent demands for proper management of DPN in patients with DM, especially patients in China.

### 2.2 Risk factors for DPN

On the other hand, the risk factors for DPN have been widely reported. Most studies have recognized that older age, longer DM duration, and worse glucose control are risk factors for DP. Meanwhile, obesity or overweight, worse renal function, unfavorable lipid profile (such as higher low-density lipoprotein cholesterol and lower high-density lipoprotein cholesterol), hypertension, and smoking are also associated with a higher risk of DPN (28, 30, 32, 33, 35, 38). Notably, two studies reported that insulin treatment is associated with a higher risk of DPN (28, 30). Interestingly, a study conducted in Saudi Arabia reported that females are more likely to suffer from DPN (30), while a study performed in the United States suggested that in youth with type 2 DM, male sex is a risk factor for DPN (30). The UK prospective diabetes study (UKPDS) revealed that age, female sex, poor glucose control, weight, alcohol consumption, and current smoking are risk factors for DPN (39). There are also several studies reporting novel factors associated with DPN risk. For instance, lower education level (26), lower vitamin D level or vitamin D deficiency (40), receiving β-blocker treatment (34), the incidence of diabetic retinopathy (41), and lower income (42).

### 2.3 Pharmacological treatments for DPN

According to the relevant guidelines in China released in 2021 (10), pregabalin and duloxetine are the first-line pharmacological treatments for painful DPN. Meanwhile, considering the economic burden and comorbidities, gabapentin could also serve as an initial therapy. Tricyclic antidepressants such as amitriptyline and imipramine should be carefully administered due to the high incidence of adverse events. Opioids such as tramadol are not recommended due to the high incidence of adverse events and addiction (10). These agents are recommended for the treatment of DPN due to their analgesic effects. Nevertheless, they could not reverse the injury of neurons in patients with DM. Therefore, it is of great significance to search for potential treatment strategies to alleviate the pathogenesis and progression of DPN.

## 3 Preclinical evidence of neuroprotective effects of GLP-1R agonists in DPN

In patients with DM, the metabolism of glucose, lipids, proteins, and nuclear acids is largely dysregulated. Under hyperglycemia, lipids, proteins, and nuclear acids can form advanced glycation end products, interact with the corresponding receptor and finally trigger the activation of inflammation, oxidative stress, and mitochondrial dysfunction, which ultimately induces neuronal injury (43–46).

### 3.1 *In vitro* evidence

Qi et al. used methylglyoxal, a byproduct of glucose metabolism, to treat neuroblastoma SH-SY5Y cells to mimic diabetic neuropathy (47). The authors then used GLP-1R agonist liraglutide to treat the methygluoxal-induced SH-SY5Y cells. Liraglutide treatment decreased the levels of superoxide dismutase (SOD) and reactive oxygen species (ROS), suggesting that liraglutide reduced oxidative stress in methygluoxal-induced SH-SY5Y cells. Moreover, the authors also used ^1^H nuclear magnetic resonance and disclosed that liraglutide altered energy metabolism and elevated gluconeogenesis; it also increased oxidative phosphorylation while suppressing glycolysis in methygluoxal-induced SH-SY5Y cells (47).

Pandey et al. used high-glucose treatment in SH-SY5Y cells to mimic diabetic neuropathy and explored the effect of the GLP-1R agonist exendin-4 on diabetic neuropathy (48). The data showed that exendin-4 treatment elevated the levels of p-protein kinase B (Akt) and B-cell lymphoma 2 (Bcl-2) and suppressed the level of Bax, which indicated a lower level of cell apoptosis. The authors also found that exendin-4 treatment inhibited the level of oxidative stress. In addition, exendin-4 treatment repressed mitochondrial dysfunction, as shown by lower levels of the mitochondrial function-associated genes MCU and UCP3, as well as the mitochondrial fission genes DRP1 and FIS1. More importantly, the authors demonstrated that exendin-4 treatment exerted the abovementioned functions through the Epac/Akt pathway (48).

### 3.2 *In vivo* evidence

Liu et al. established a DM rat model by injection of streptozotocin; then, they treated the DM rats with intraperitoneal injection of exendin-4 at 1 nmol/kg for 24 weeks (49). The authors found that compared with placebo injection, the 2000 and 250 Hz current perception threshold values were reduced by exendin-4 injection, indicating a lower level of nerve dysfunction. They also revealed that the reduction in the axon/fibre area ratio of the sciatic nerve and intraepidermal nerve fibre loss were relieved by exendin-4 injection (49).

Jolivalt et al. treated streptozotocin-induced DM rats with exenatide, a GLP-1R agonist, for 8 weeks and found that exenatide treatment attenuated the reduction in motor nerve conduction velocity and paw intraepidermal fibre density (50). The authors also elucidated that exenatide elevated phosphorylation of extracellular signal-regulated kinases 1/2 (ERK1/2) level, suggesting that exenatide exerted a neuroprotective effect through activating the ERK signaling. Interestingly, the authors also found that exenatide did not vary the blood glucose, insulin level, or body weight in streptozotocin-induced DM rats. The findings of the authors suggested that the neuroprotective effect of exenatide was independent of the glycemic control effect of exenatide (50).

Himeno et al. dissected GLP-1R-expressing lumbar dorsal root ganglion neurons from mice and cultured them with Schwann cell-conditioned media to mimic diabetic conditions (51). The authors then treated dorsal root ganglion neurons with exendin-4. The data revealed that exendin-4 significantly promoted the neurite outgrowth that was previously impaired by Schwann cell-conditioned media. In the *in vivo* experiments, the authors treated the streptozotocin-induced DM mice with exendin-4 at 10 nmol/kg for 4 weeks and found that exendin-4 promoted the peripheral nerve function as reflected by improved current perception threshold and motor and sensory nerve conduction velocity. Interestingly, the authors revealed that the dose of exendin-4 at 10 nmol/kg for 4 weeks had no effect on blood glucose, body weight, or HbA1c levels in streptozotocin-induced DM mice. These findings indicated that the neuroprotective effect of exendin-4 was independent of its blood glucose-lowering effect (51).

Ma et al. also used streptozotocin injection to establish a DM rat model (22). Subsequently, the daily GLP-1R agonist liraglutide at 200 μg/kg was used to treat the DM rats for 8 weeks. The authors observed that the loss of myelin nerve fibre density was partly restored by liraglutide treatment, and the nerve conduction velocity of motor and sensory nerves was improved by liraglutide treatment. The authors also noticed that the levels of proinflammatory cytokines (including cytokines tumor necrosis factor-α (TNF-α), interleukin-1β (IL-1β), and IL-6), intercellular adhesion molecule 1 (ICAM1), and NADPH oxidase 4 (NOX4)) in the sciatic nerve were all reduced by liraglutide treatment. The authors further explored the relevant signaling pathways and found that mitogen-activated protein kinase (MAPK) and nuclear factor-κB (NF-κB) were repressed by liraglutide treatment (22).

Moustafa et al. established a DM rat model by injecting streptozotocin and nicotinamide (21). Then, they treated DM rats with liraglutide or pregabalin, a therapeutic agent for neurological pain in patients with DPN. The data showed that both liraglutide and pregabalin showed similar effects on the neurological behaviors of DM rats as assessed by motor coordination and latency withdrawal time of tail flick and hind paw cold allodynia tests. Meanwhile, liraglutide ameliorated the dysregulation of malondialdehyde (MDA), nitric oxide (NO), SOD, matrix metalloproteinase (MMP)-2 and -9, as well as IL-6 and IL-10 in the sciatic nerve, while pregabalin had less effect on them. These findings suggested that liraglutide may have similar effects on ameliorating neurological symptoms of DPN as pregabalin, and it also mitigated oxidative stress, inflammation, and matrix remodelling in DPN (21).

**Figure 1:**
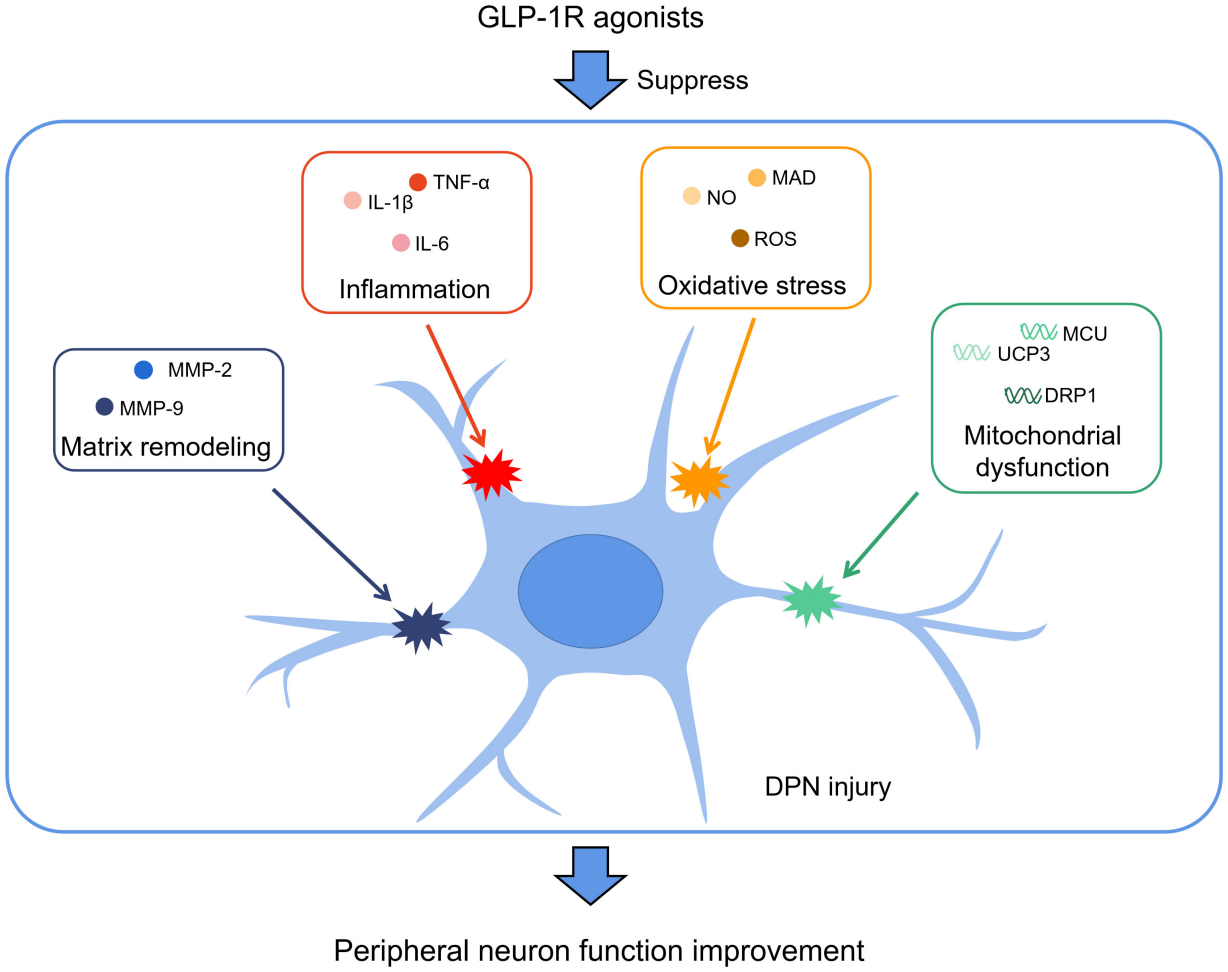
GLP-1R agonists reduce inflammation, oxidative stress, mitochondrial dysfunction, and matrix remodelling to improve neuron functions.

Apart from the abovementioned studies, there are also several studies demonstrating the neuroprotective effects of GLP-1R agonists in DM, and we have listed these studies (including the abovementioned studies) in **Table 1**. Combined with the above-described studies, it is clear that GLP-1R agonists reduced inflammation, oxidative stress, mitochondrial dysfunction, and matrix remodelling to improve neuron functions in DPN (**Figure 1**). Notably, two studies controlled the dosage of GLP-1R agonists at a level that would not affect the blood glucose of *in vivo* DM models, and these two studies demonstrated that the neuroprotective effects of GLP-1R agonists are independent of their blood glucose-lowering effects. The molecular mechanisms of the neuroprotective effects of GLP-1R agonists include signaling pathways such as ERK signaling, PI3K signaling, RhoA activity, MAPK/NF-κB signaling, and the Epac/Akt pathway. These studies have laid a solid basis for the clinical application of GLP-1R agonists for treating DPN.

**Table 1:** Preclinical studies exploring the neuroprotective effects of GLP-1R agonists.

Author	Model	GLP-1R agonists	Findings
*In vitro* evidence			
Tsukamoto et al. (52)	Insulin removed medium-cultured rat dorsal root ganglion neurons	Exendin-4	Exendin-4 promoted neurite outgrowth and viability of dorsal root ganglion neurons through activation of PI3K signaling.
Mohiuddin et al. (20)	Hydrogen preoxide-treated dorsal root ganglion neurons	Exendin-4	Exendin-4 reduced oxidative stress and promoted neurite projection.
Kornelius et al. (53)	High glucose and high free fatty acid-treated RSC96 Schwann cells	Liraglutide	Liraglutide increased cell viability, reduced oxidative stress, inhibited inflammation, and upregulated neurotrophic factors.
Qi et al. (47)	Methylglyoxal-treated SH-SY5Y cells	Liraglutide	Liraglutide repressed oxidative stress and promoted gluconeogenesis
Pandey et al. (48)	High glucose-treated SH-SY5Y cells	Exendin-4	Exendin-4 inhibited cell apoptosis, suppressed oxidative stress, and relieved mitochondrial dysfunction through Epac/Akt signaling
*In vivo* evidence			
Liu et al. (49)	Streptozotocin-induced diabetic rats	Exendin-4	Exendin-4 promoted neuron functions.
Jolivalt et al. (50)	Streptozotocin-induced diabetic mice	Exenatide	Exenatide did not affect blood glucose and promoted neuron functions.
Himeno et al. (51)	Schwann cell conditioned media-cultured GLP-1R-expressing dorsal root ganglion neurons	Exendin-4	Exendin-4 promoted neurite outgrowth of dorsal root ganglion neurons.
	Streptozotocin-induced diabetic mice		Exendin-4 did not affect blood glucose and promoted neuron functions.
Gumuslu et al. (54)	Streptozotocin/nicotinamide-induced diabetic mice	Exenatide	Exenatide promoted hippocampal genetic level of GLP-1R and nerve growth factor
Ma et al. (22)	Streptozotocin-induced diabetic rats	Liraglutide	Liraglutide elevated neuron function, reduced inflammation, and suppressed p-p38 MAKP/NF-κB signaling
Moustafa et al. (21)	Streptozotocin/nicotinamide-induced diabetic rats	Liraglutide	Liraglutide increased animal behavior and inhibited oxidative stress, inflammation, and matrix remodelling.
Zhang et al. (55)	Streptozotocin-induced diabetic mice	Liraglutide	Liraglutide relieved neuropathic pain and inhibited cortical microglia activation.

GLP-1R, glucagon-like peptide-1 receptor; PI3K, phosphoinositide 3-kinase; MAKP, mitogen-activated protein kinase; NF-κB, nuclear factor-κB; Akt: protein kinase B.

## 4 Clinical evidence of GLP-1R agonists in managing DPN

Unlike preclinical evidence, studies investigating the effect of GLP-1R on DPN are quite rare. Most studies reported diabetic neuropathy as one of the endpoints, rather than classifying it into peripheral neuropathy and autonomic neuropathy. A brief description of the studies is listed in **Table 2**.

**Table 2:** Clinical evidence of GLP-1R agonists for treating DPN.

Author	Design	Patients	Grouping	Treatment duration of Randomized, controlled trial	Neurological findings
Sullivan et al. (56)	Randomized, controlled trial	Type 2 DM	Liraglutide 1.2 mg/day (N=251) Liraglutide 1.8 mg/day (N=247) Glimepiride 8 mg/day (N=248)	52 weeks	Neuropathies leading to first or recurrent amputation was higher for glimepiride compared to both doses of liraglutide.
Jaiswal et al. (23)	Randomized, controlled trial	Type 2 DM	Exenatide (N=22) Insulin glargine (N=24)	18 months	No difference was found in confirmed clinical neuropathy, intraepidermal nerve fibre density, or findings of nerve conduction studies between groups.
Brock et al. (57)	Randomized, controlled trial	Type 1 DM with DPN	Liraglutide (N=19) Placebo (N=20)	26 weeks	No difference was found in neuronal function between groups.
Ponirakis et al. (58)	Randomized, controlled trial	Poorly controlled type 2 DM	Exenatide plus pioglitazone (N=21) Aspart insulin with glargine (N=17)	1 year	Corneal nerve branch density was increased, but vibration perception was worsened in exenatide plus pioglitazone group after treatment.
Issar et al. (59)	Cohort study	Type 2 DM	Exenatide (N=32) DDP-4 inhibitor (N=31) SGLT-2 inhibitor (M=27)	–	Exenatide was associated with an improvement in nerve function.
Lin et al. (60)	Cohort study	DM	GLP-1R agonists (N=20,288) SGLT-2 inhibitor (N=81,152)	–	GLP-1R agonists usage was associated with lower risk of major adverse limb events (including nontraumatic amputation), especially in patients with DPN.

GLP-1R, glucagon-like peptide-1 receptor; DPN, diabetic peripheral neuropathy; DM, diabetes mellitus; DDP-4, dipeptidyl peptidase-4; SGLT-2, sodium-glucose cotransporter-2.

Sullivan et al. conducted an analysis of the long-term effectiveness of liraglutide and glimepiride monotherapies in patients with DM from the LEAD-3 trial (56). The LEAD-3 trial assigned patients to randomly receive monotherapies of liraglutide 1.2 mg/day, liraglutide 1.8 mg/day, and glimepiride 8 mg/day (61). The analysis of Sullivan et al. revealed that the rates of neuropathies leading to first/recurrent amputation within 30 years were higher in patients receiving glimepiride 8 mg/day (N=248) than in patients receiving liraglutide 1.2 mg/day (N=251) or liraglutide 1.8 mg/day (n=247), while they were numerically similar between patients receiving liraglutide 1.2 mg/day and liraglutide 1.8 mg/day. Meanwhile, the incidence of minor hypoglycemia was higher in patients receiving glimepiride 8 mg/day than in those receiving liraglutide 1.2 mg/day or liraglutide 1.8 mg/day (56). The findings of this study disclosed that liraglutide showed a better effect on treating DPN. Nevertheless, the main populations of this study are Caucasian, Hispanic, and African-American. Therefore, the findings of this study could not be expanded in Asia. In addition, the sample size of this study is not large enough to drive a solid conclusion. Meanwhile, the evaluation of neuropathy was not mentioned in this article.

In a pilot study, Jaiswal et al. enrolled 46 type 2 DM patients with mild to moderate DPN (23). The 46 patients were randomized to receive open-label twice-daily exenatide (N=22) or daily insulin glargine (N=24). The primary outcome was the prevalence of confirmed clinical neuropathy assessed by nerve conduction studies of the median, peroneal motor, and sural sensory nerves with a standard protocol. The secondary outcomes included individual electrophysiology measures, vibration perception, clinical neuropathy changes, etc. After 18 months of follow-up, blood glucose control was similar between the groups. Meanwhile, the prevalence of confirmed clinical neuropathy, intraepidermal nerve fibre density, and nerve conduction were all similar between groups. Nevertheless, limited by the small sample size and the open-label study design, the findings may be biased. In addition, the abnormal nerve conduction study measures at baseline were slightly different (although not statistically significant); this could also affect the findings (23).

Brock et al. conducted a randomized, double-blinded, placebo-controlled trial to explore the effect of liraglutide on neuropathies in patients with type 1 DM (57). The patients in the liraglutide group received 1.2-1.8 mg daily liraglutide for 26 weeks. The primary outcome was the change in latency of early brain evoked potentials. Secondary outcomes included cortical evoked potential, peripheral neurophysiological testing, proinflammatory cytokines, and autonomic function. Peripheral nerve function was assessed by nerve conduction velocities, amplitudes and F‐waves on the motor and sensory nerves. The data showed that liraglutide significantly decreased the level of IL-6 and numerically reduced the levels of other proinflammatory cytokines, including interferon-γ and IL-10. However, the functions of central, autonomic, or peripheral neurons were not affected by liraglutide treatment (57). This study could still be limited by the small sample size. In addition, all patients had type 1 DM, and the neuroprotective effect of liraglutide in patients with type 2 DM was unclear.

Ponirakis et al. conducted a subgroup analysis of the Qatar study, an open-label, randomized controlled trial (62), and compared the peripheral neurological outcomes in patients receiving a combination of exenatide (2 mg/week) and pioglitazone (30 mg/day) and those receiving glargine plus aspart insulin (58). The primary outcome was the findings of corneal confocal microscopy assessing small nerve fibres. The secondary outcomes were vibration perception threshold assessed by the Neurothesiometer, sudomotor function evaluated by electrochemical skin conductance, and neuropathic pain assessed by the Douleur Neuropathique en 4 questionnaire. The authors disclosed that compared with baseline, the combination treatment group (N=21) showed an increase in corneal nerve branch density but worsened vibration perception and unchanged sudomotor function as well as neuropathic pain after 1 year of treatment. Inheriting from the main study, this subgroup analysis has the limitations of small sample size and open-label study design (58).

Issar et al. reported an observational comparative study that compared nerve excitability in patients with type 2 DM who received exenatide (n=32), a dipeptidyl peptidase-4 (DPP-4) inhibitor (n=31), or a sodium-glucose cotransporter-2 (SGLT-2) inhibitor (n=27) (59). Motor nerve excitability was assessed by the function of voltage-gated sodium and potassium ion channels and sodium-potassium pumps. The authors disclosed that in patients who received a DPP-4 inhibitor or an SGLT-2 inhibitor, abnormalities were found in peak threshold reduction, S2 accommodation, and subexcitability and superexcitability, whereas patients who received exenatide showed normal nerve excitability. Moreover, the authors found that exenatide treatment was associated with elevation of nerve function, which was independent of blood glucose control (59). Although these findings were encouraging, further randomized, controlled trials are needed to verify them.

In a retrospective cohort study, Lin et al. (60) reviewed patients with DM who received GLP-1R agonists (n=20,288) and SGLT-2 inhibitors (n=81,152). The primary outcome was the incidence of major adverse limb events (defined as either one of the following events: newly diagnosed critical limb ischemia, percutaneous transluminal angioplasty or peripheral bypass of peripheral artery disease, or nontraumatic amputation). The secondary outcome was major adverse cardiovascular events. The incidence of major adverse limb events was lower in patients who received GLP-1R agonists than in those who received an SGLT-2 inhibitor. In addition, the association of GLP-1R agonists with the low incidence of major adverse limb events was notable in patients with DPN. In fact, DPN is closely associated with adverse limb events. For instance, a *post hoc* analysis of the Action to Control Cardiovascular Risk in Diabetes (ACCORD) trial illustrated that DPN was a strong predictor of lower-limb amputations (63). Meanwhile, DPN is independently associated with the risk of diabetic foot ulcers, which could lead to nontraumatic lower-limb amputations (64). Although the findings of Lin et al. did not directly point out the treatment potential of GLP-1R agonists for DPN, these results may support the peripheral neuroprotective effect of GLP-1R agonists considering the close association of DPN with adverse limb events (60).

Based on these studies, the treatment efficacy of GLP-1R agonists in patients with DPN is still controversial. In addition, most studies are limited by small sample sizes. On the other hand, only a few studies clearly reported DPN rather than combining it with autonomic neuropathy. Moreover, the assessments of neurological function varied greatly among studies, which may also lead to inconsistent findings among studies. Therefore, further large-scale studies are needed to further explore the therapeutic efficacy of GLP-1R agonists in patients with DPN. Meanwhile, since this study is not a meta-analysis, the association of GLP-1R agonists with outcomes of patients with DPN could not be fully addressed.

## 5 Conclusion

In conclusion, the incidence of DPN varies from nearly 20% to over 70% in patients with DM, which severely hampers quality of life and even leads to amputation. The currently recommended pharmacological treatments for DPN mainly include antidepressants due to their analgesic effects, while a strategy for the pathogenesis and progression of DPN is lacking. GLP-1R agonists exert neuroprotective effects in DPN by reducing neuronal apoptosis, inflammation, oxidative stress, mitochondrial dysfunction, and matrix remodelling. Nevertheless, the available clinical evidence on the neuroprotective effects of GLP-1R agonists in DPN is still controversial and limited. Therefore, it is urgent to validate the therapeutic effect of GLP-1R agonists with large-scale studies and well-designed randomized, controlled trials. Additionally, systematic review and meta-analysis would be helpful to address the role of GLP-1R agonists in treating DPN.

## Author contributions

CL: Conceptualization, Formal Analysis, Writing – original draft, Writing – review & editing. TW: Formal Analysis, Writing – review & editing. NR: Conceptualization, Funding acquisition, Resources, Writing – review & editing.
